# Evaluating Custom and Transfer Learning Convolutional Neural Networks for Pneumonia Classification Under Data Scarcity and Low-Compute Conditions for Under-Resourced Environments

**DOI:** 10.7759/cureus.97959

**Published:** 2025-11-27

**Authors:** Andy L Chen, Eric Cheek, Morteza Samradi

**Affiliations:** 1 Computer Science, Monta Vista High School, Cupertino, USA; 2 Electrical Engineering and Computer Science, University of Michigan, Ann Arbor, USA; 3 Research, Massachusetts Institute of Technology, Cambridge, USA

**Keywords:** cnn, deep learning, low-resource environments, pneumonia classification, raspberry pi, transfer learning

## Abstract

Pneumonia is an infectious disease that causes many deaths across the world. By accurately and efficiently recognizing pneumonia from images using machine learning, we can facilitate faster treatment. Yet, in under-resourced environments, there is often a lack of training data, high-quality images for training, as well as a lack of high computing power. As such, we aim to investigate and address these issues. In this paper, we deployed and tested different convolutional neural networks (CNNs), optimizing them to use fewer resources. We simulated the low training image count, low image quality, and low compute power, using a Raspberry Pi and the Pneumonia Modified National Institute of Standards and Technology (MNIST) dataset with 64x64 images. We were able to achieve accuracies of 95% for our laptop-trained, quantized model and 94% for our P100 quantized model. The F1 scores, as well as the recall for the pneumonia class, were also evaluated to provide additional insights into the performance of the models. Furthermore, we were able to obtain a rounded average latency of 1.46 milliseconds (ms) for our P100 quantized model, with a rounded model size of 1.45 megabytes (MB). Our work allows for an accurate diagnosis of pneumonia with limited resources.

## Introduction

Machine learning in the medical field has many useful applications, such as disease classification and image denoising. Machine learning for disease classification is especially important, allowing for better and potentially faster diagnosis of certain diseases [1].

Being the largest infectious cause of death in children worldwide, pneumonia is an acute respiratory infection, where the small sacs in the lungs become filled with fluid, causing breathing difficulty [2]. Often diagnosed with a chest X-ray, early detection of pneumonia is an important step in ensuring fast treatment and a higher chance of survival. Since 2018, more than 40,000 people a year have died of pneumonia in the United States, and it accounts for 14% of all deaths in children under five, killing 740,180 children in 2019 [2-4].

However, while many hospitals have access to advanced technologies and high-quality images, under-resourced hospitals face more issues. Many research papers have investigated how convolutional neural networks (CNNs) can be used to classify images, but there is a lack of research for under-resourced environments and hospitals [5]. While training models can be done off-site on a powerful graphics processing unit (GPU), many hospitals themselves do not have access to them when actually diagnosing or performing inference. Additionally, many hospitals lack powerful equipment to produce high-quality X-ray images, and/or cannot produce as many training images as they would like. As such, even if trained on high-quality images, models may perform poorly when actually deployed in those hospitals [6].

Therefore, in our research, we used a Raspberry Pi 5 to simulate the low computing power, 64 x 64 image quality to simulate the low image quality, and less than 6000 images to simulate the low training image count. Additionally, many research papers investigate transfer learning models, which are often larger and can lead to higher inference times (latency) and model size [7]. Therefore, in this paper, we focus especially on custom-built CNNs with minimal layers to optimize for performance. 

In this study, we hope to identify how we can build a CNN that is both lightweight and can classify pneumonia accurately under resource-constrained settings, such as low compute power and low-quality images. We will compare the metrics such as accuracy and F1 scores of multiple models, with and without quantization, to evaluate both the efficiency and the classification performance of the model. 

Related research on the diagnosis of pneumonia 

Many other papers have investigated the diagnosis of pneumonia with machine learning. Some have also investigated it in the context of under-resourced environments. In a 2020 study, Cococi et al. [8] discussed and investigated various transfer models for the classification of pneumonia (unmodified and modified) like MobileNetV3 [9] variations, ShuffleNetV2 [10], and L-CNN, a trainable version of Binary Weights Convolutional-Extreme Learning Machine (BCONV-ELM) [11]. They trained on a laptop with a GPU, testing unquantized versions of the models on a Raspberry Pi 4, and quantized versions on an Android device. They scaled down their images to 224 x 224 pixels, and used data augmentation to go from 5856 images to 17470 total images. For two-class classification on a Raspberry Pi 4 with unquantized models, they were able to obtain an accuracy of 96.12% for one model, and a latency of 30 ms for another model. On an Android device with quantization, they obtained an accuracy of 94.44% on one model, and a latency of 7 ms on another model. The model that achieved the highest accuracy on the Raspberry Pi 4 had a latency of 167 ms with a model size of 35 MB (unquantized original model). The model that achieved the highest accuracy on the Android device had a latency of 32 ms with a size of 551 kilobytes (KB) after quantization. We aim to expand on this by testing custom CNNs, along with lower image quality and using quantized versions of models. We only focused on testing on a Raspberry Pi for our research. 

In a 2024 study, Kiche et al. [12] investigated a transfer learning model, DenseNet121 [13]. They focused on four class classification using a total of 7135 images. The study discussed and found issues related to things such as class imbalances and used data augmentation to find a solution. The evaluation found F1 scores of 0.51 and 0.85 for the normal and pneumonia classes, respectively, and obtained an accuracy of 0.98 for the normal class and a recall of 0.99 for the pneumonia class. The relatively low F1 scores indicated that some classes may have either low recall or low precision, or a mix of both. In the context of pneumonia classification in medical imaging, the recall for any class that is not normal is highly important, and should be evaluated alongside the overall accuracy. In our research, we hope to improve upon these results by using a custom CNN and only two classes. We aim to maintain the high recall for pneumonia while improving the overall accuracy as well as the recall for the normal class. 

## Materials and methods

Dataset and preprocessing 

**Figure 1:**
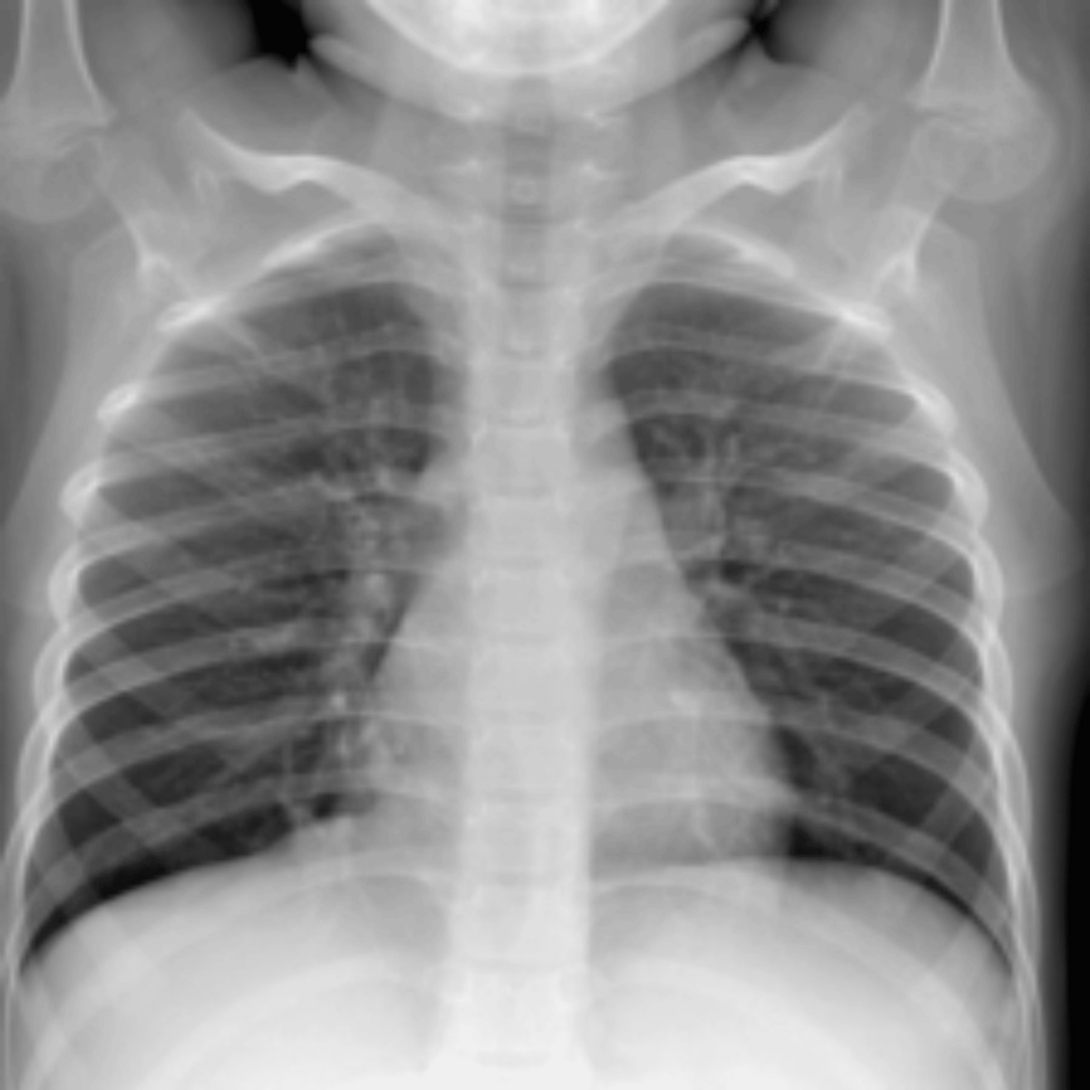
Image of a normal lung from the pneumonia MNIST dataset MNIST: Modified National Institute of Standards and Technology. Please note that this is a higher-quality image from the 224 x 224 resolution dataset that has also been rescaled to a resolution of 900 x 900. We used lower-quality images from the 64 x 64 resolution dataset. The images are otherwise the same across the two datasets.

**Figure 2:**
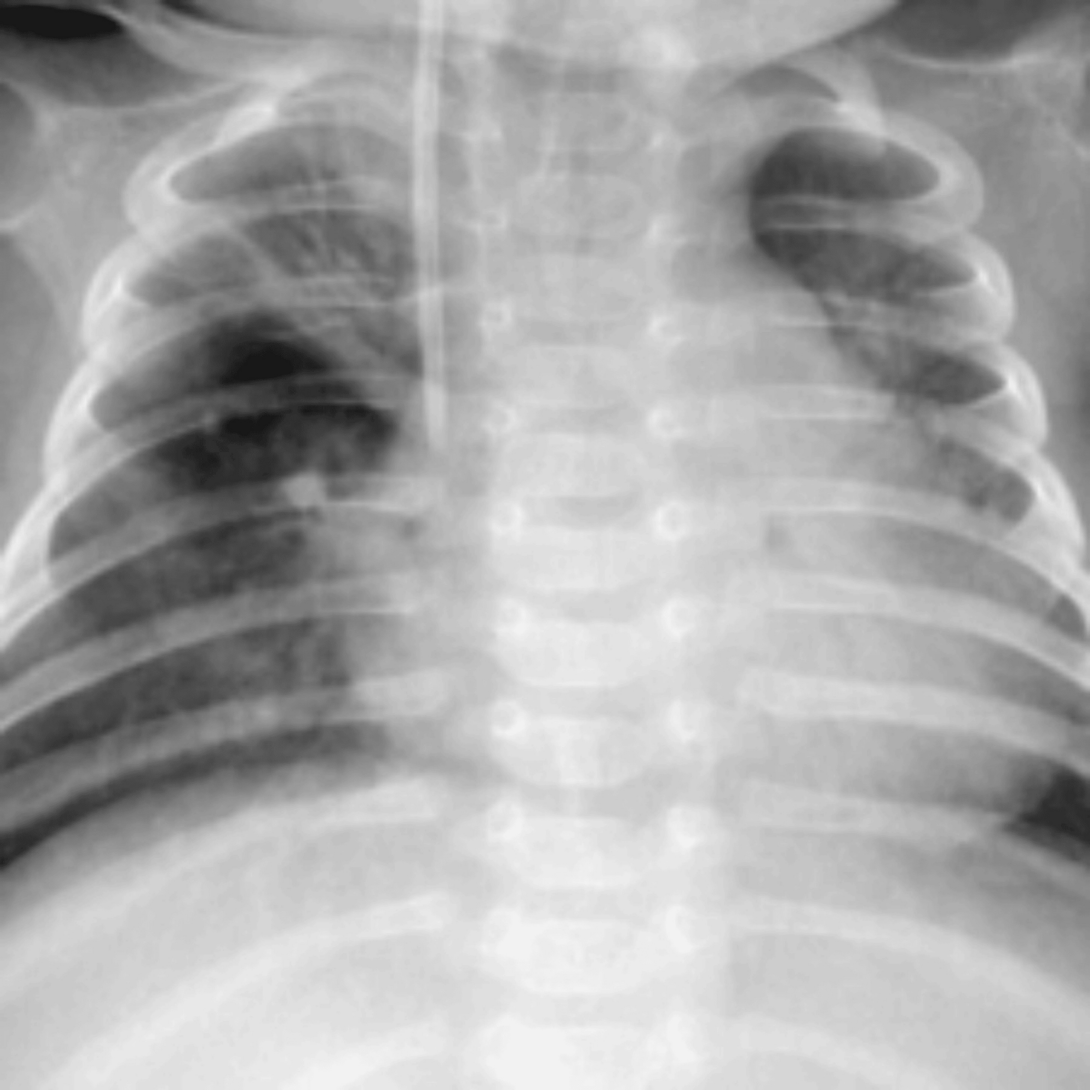
Image of a lung with pneumonia from the pneumonia MNIST dataset MNIST: Modified National Institute of Standards and Technology. Please note that this is a higher-quality image from the 224 x 224 resolution dataset that has also been rescaled to a resolution of 900 x 900. We used lower-quality images from the 64 x 64 resolution dataset. The images are otherwise the same across the two datasets.

For our dataset, we specifically chose the Pneumonia MNIST 64 x 64 dataset [14], with 4708 training images, 624 testing images, and 524 validation images, all in 64 x 64 resolution/size. The 64 x 64 dataset had a size of 20.6 MB. The dataset contained two classes for binary classification: images with healthy (normal) lungs, and ones with pneumonia. Figures 1, 2 show a healthy and diseased (pneumonia) lung, respectively. 

Images such as these were used to train our models to help them distinguish between normal X-rays and those representing pneumonia. 

To normalize the pixel values, we divided all the values by 255.0 to scale them from 0 to 1. This helped to improve model training stability, as the inputs were smaller. While the images were all grayscale, we added a channel dimension at the end with a value of 1 to allow our model to correctly process the grayscale images, as most neural networks for images expect a channel dimension. 

Because we had an imbalanced dataset and a low amount of training images to begin with, we used data augmentation to artificially create images for the normal class. This process allows us to artificially create more images for the normal class, which can improve the model's accuracy for that category. This helps to address issues such as the recall for one class being much higher than the other. Before augmentation, there were 1214 images for the normal class and 3494 images for the pneumonia class in the training set. After augmentation, there were 15782 images for the normal class and 3494 images for the pneumonia class. In doing so, we were able to improve the recall for the normal class and overall accuracy.

We used a variety of methods to augment the images. They were first randomly cropped to 54 x 54 size, then resized back to the original 64 x 64 size. Next, we did the following randomly: flipped the image left and right, changed the contrast, rotated it, added noise, and then clipped all values back between 0.0 and 1.0 to ensure the normalization of the pixel values.

To ensure consistency, the random seeds and other sources of randomness were all set, using a random seed of 42.

Model architecture

We tested a total of 12 models in our research. For the purpose of this research, the transfer models (six of them) were not tuned or optimized extensively, and were only tested in order to compare model sizes and latencies between the transfer models and the custom-built CNNs.

The six transfer models are as follows: Visual Geometry Group (VGG16) model [15] without and without quantization, MobileNetV2 with and without quantization [16], and EfficientNetB0 [17] with and without quantization. 

The six custom built CNNs were all constructed using the same code, but were trained on different GPUs. While randomness was controlled as much as possible by setting random seeds and other sources of randomness in advance, the exact same methods for training code still yielded a slightly different model and results. Therefore, the six custom-built CNN models were as follows: P100-CNN, P100-CNN-Quantized, Laptop-CNN, Laptop-CNN-Quantized, T4-CNN, T4-CNN-Quantized. Each CNN model name corresponds to the specific GPU it was trained on: Kaggle's P100 GPU (Nvidia Corporation, California, US), an Nvidia GeForce RTX 4050 Laptop GPU, and Kaggle's 2 x T4 GPUs. The quantized at the end of the CNN model name indicates it was converted to a Tensorflow-Lite model with quantizations, using the default optimizations from TensorFlow. The CNN model names without the term 'quantized' at the end indicate that the model was converted to a Tensorflow-Lite without any quantizations or optimizations. Each GPU yielded a model with slightly different size, latency, and result. 

**Figure 3:**
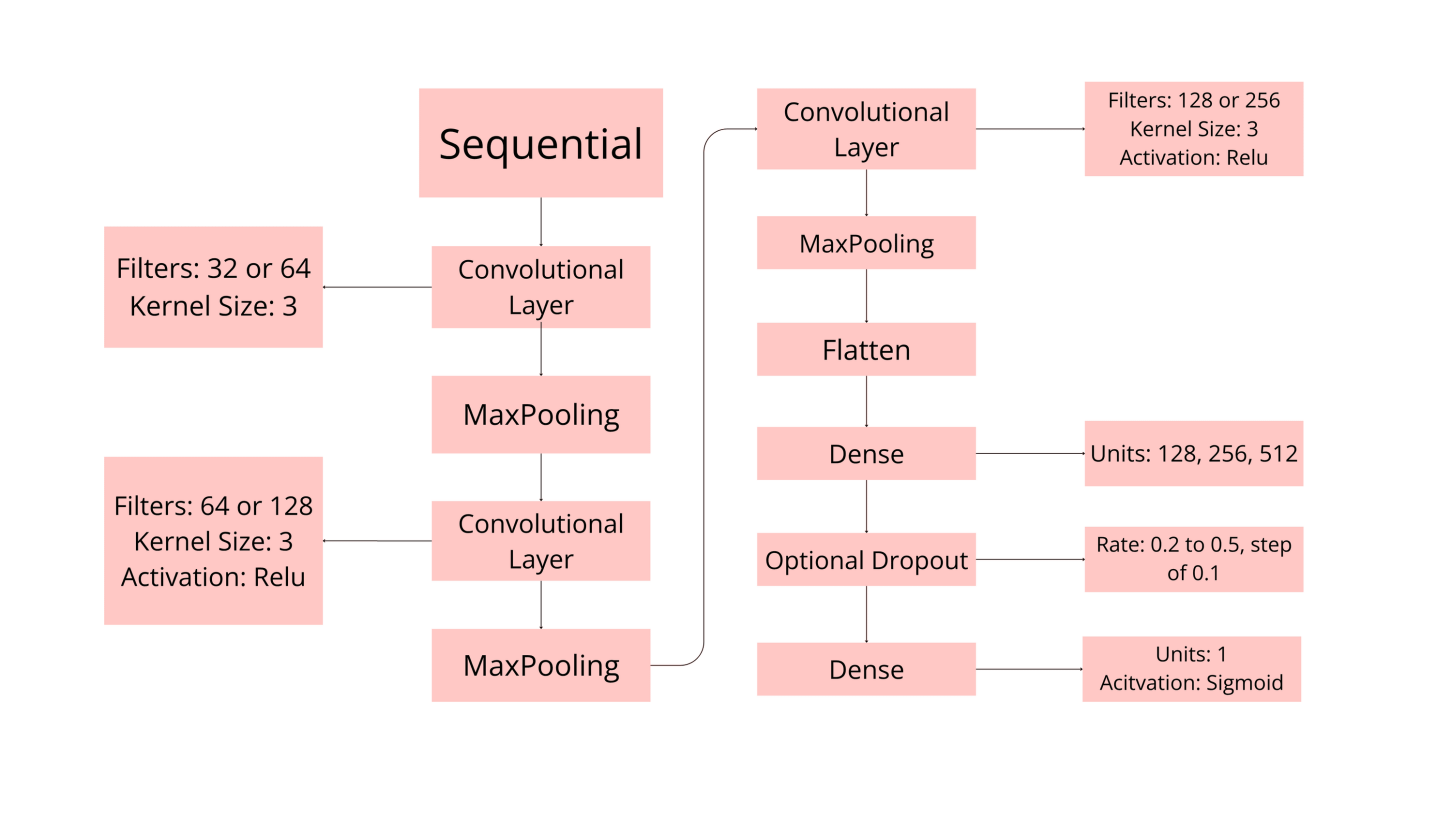
Flowchart of model architecture Model Architecture Flowchart, visualizing the layers as well as the possible hyperparameters for each layer that the keras-tuner tuned over. Figure created by Chen A using Canva (Canva Pty Ltd., Sydney, Australia).

The custom CNN was built using a Keras Sequential model. It consists of two convolutional layers followed by two dense layers, with each layer using a Rectified Linear Unit (ReLU) activation except the last, with the last dense layer using a sigmoid activation for binary classification. To determine the best hyperparameters, we used a keras-tuner to select them. The keras-tuner was validated on the dataset's existing validation data. The testing set was kept out from model selection and tuning, and was only used at the end for the final evaluation of model performance. We used the keras-tuner's RandomSearch with 60 max trials, one execution per trial, with the objective to find the model build that maximizes validation accuracy. The tuner then searched with 30 epochs, an EarlyStopping callback with patience of five, a Reduce Learning Rate On Plateau callback (factor of 0.5 and patience of two), and a batch size of 32. Figure 3 shows the specific model architecture along with the hyperparameter choices that the keras-tuner tested.

Table 1 shows the specific hyperparameters for each custom model.

**Table 1:** Final hyperparameters selected by the keras-tuner for each custom model CNN: Convolutional neural networks.

Hyperparameter	Laptop-CNN	T4-CNN	P100-CNN
Conv Layer 1 Filters	32	32	32
Conv Layer 2 Filters	128	128	128
Conv Layer 3 Filters	256	256	256
Dense Units	512	128	128
Use Dropout	False	False	False
Learning Rate	4.481e-4	1.028e-3	1.028e-3
Dropout Rate	0.2	0.2	0.2

Because none of the models used dropout, the dropout rate is irrelevant. 

**Figure 4:**
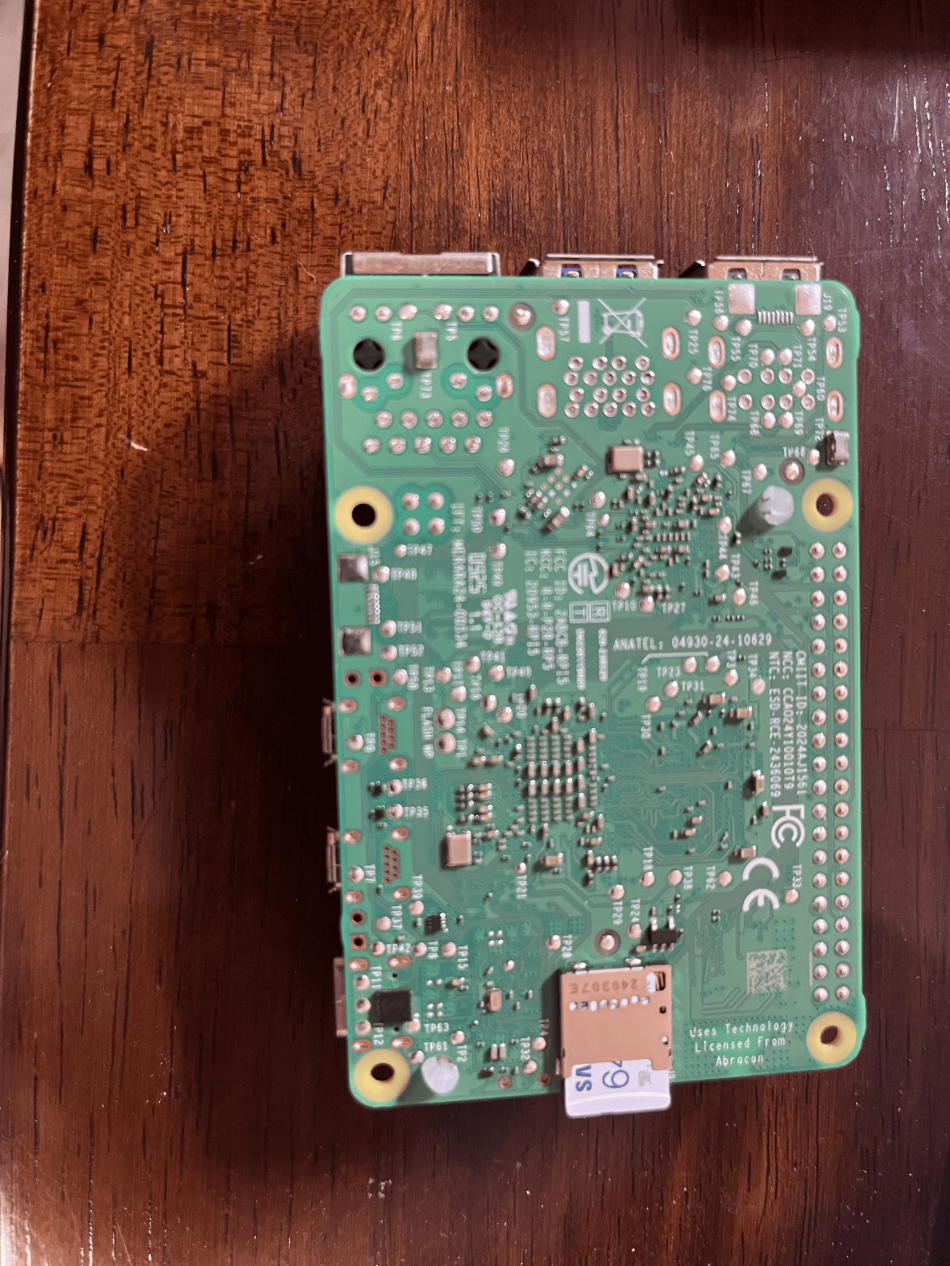
Bottom view of a Raspberry Pi 5 Picture of the Raspberry Pi used in the research, taken by Chen A.

**Figure 5:**
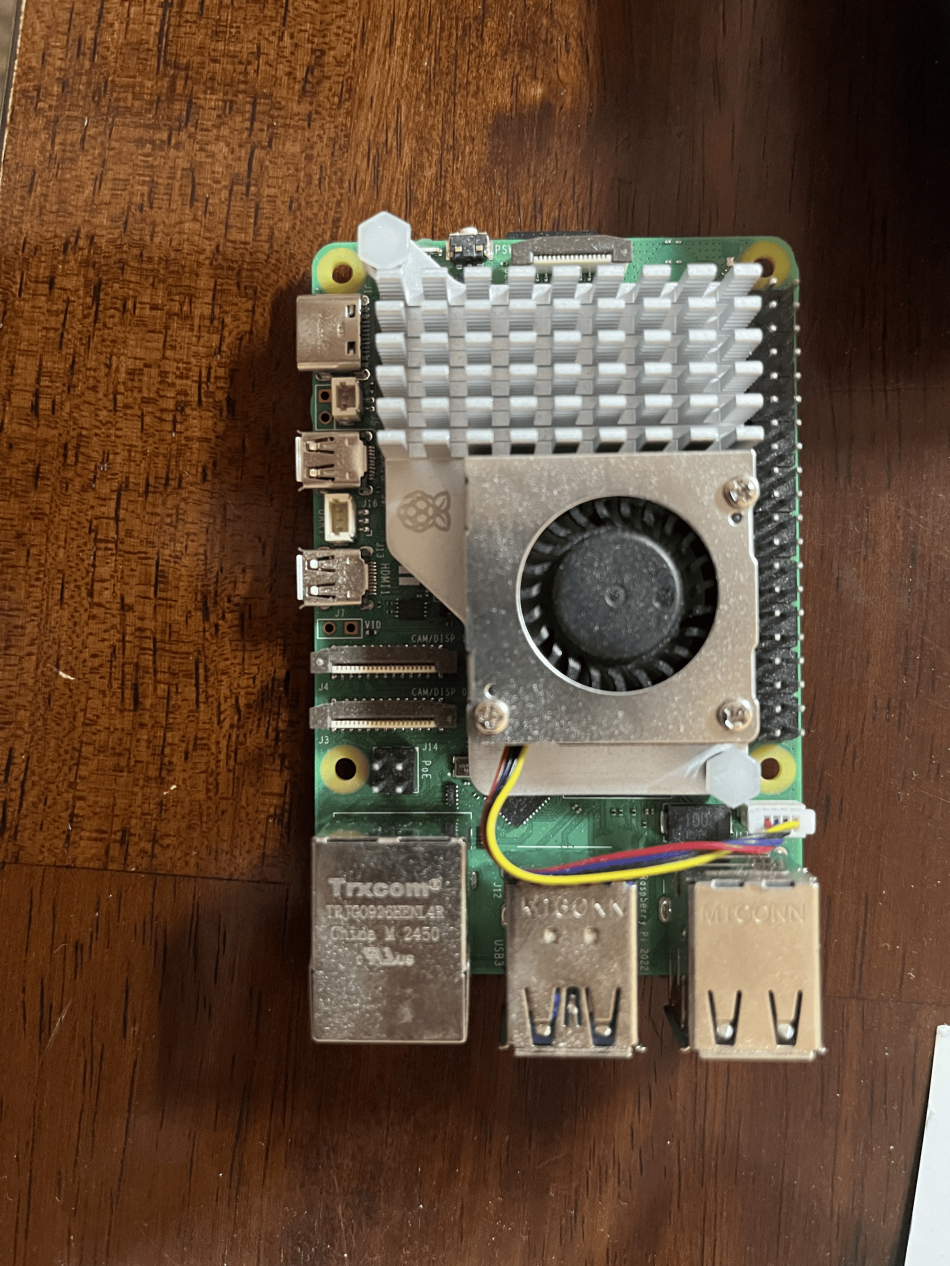
Top view of a Raspberry Pi 5 Picture of the Raspberry Pi used in the research, taken by Chen A.

As for picking the transfer models, we aimed to pick at least one small and efficient model, and one larger model. For the hardware, we tested it on a Raspberry Pi 5 (Raspberry Pi Ltd., Cambridge, UK; Figures 4, 5 show the bottom and top views, respectively). 

The Raspberry Pi we used had 16 gigabytes (GB) of random access memory (RAM), and used a 2.4GHz quad-core 64-bit Arm Cortex-A76 CPU (Arm Holdings, Cambridge, UK). As seen in the figures, the Raspberry Pi 5 is much smaller than a traditional computer and also lacks a GPU, which significantly lowers its computing and graphics processing power. In order to store the preprocessed images and data, a 64 GB Samsung EVO+ Micro SD Card (Samsung Electronics, Suwon, South Korea) was used, which also contained the Raspberry Pi Operating System.

## Results

Table 2 shows each model along with its size.

**Table 2:** Model sizes CNN: Convolutional Neural Networks; VGG: Visual Geometry Group.

Model	Size (MB)
Laptop-CNN	19.28
Laptop-CNN-Quantized	4.83
P100-CNN	5.77
P100-CNN-Quantized	1.45
T4-CNN	5.77
T4-CNN-Quantized	1.45
VGG16	56.13
VGG16-Quantized	14.09
MobileNetV2	8.41
MobileNetV2-Quantized	2.34
EfficientNetB0	15.20
EfficientNetB0-Quantized	4.24

As observed, the custom-built CNNs were generally smaller than the transfer models. Additionally, quantization was able to reduce the model size by almost four times for some of the models. For example, the Laptop-CNN is 19.28 MB before quantization, but is only 4.83 MB after quantization. Additionally, the smallest custom model is 1.45 MB for P100-CNN-Quantized, while the smallest transfer model is 2.34 MB for the MobileNetV2-Quantized.

Table 3 shows the results from the inference on the testing set on a Raspberry Pi 5.

**Table 3:** Model evaluation results on the Raspberry Pi CNN: Convolutional Neural Network. The model name along with the accuracy, pneumonia class recall, F1 scores (both normal and pneumonia class), and average latency of that model are shown. The values from this table were collected from testing done on the Raspberry Pi 5 in the research.

Model	Accuracy	Pneumonia class recall	F1 score (normal class)	F1 score (pneumonia class)	Average latency (milliseconds)
Laptop-CNN	0.95	0.97	0.93	0.96	5.71
Laptop-CNN-Quantized	0.95	0.97	0.93	0.96	1.71
P100-CNN	0.94	0.97	0.92	0.95	4.67
P100-CNN-Quantized	0.94	0.97	0.92	0.95	1.46
T4-CNN	0.91	0.98	0.87	0.93	4.67
T4-CNN-Quantized	0.91	0.98	0.87	0.93	1.46

We chose the following metrics: accuracy, recall for the pneumonia class, and latency. We chose accuracy as a general measure of how well the model performed. Recall for the pneumonia class was chosen as an important metric because it reported how many were caught out of all the pneumonia images. This is especially important in the context of medical imaging, as recognizing as many diseased images as possible should be prioritized. Latency was chosen because it measured how fast a model can perform inference, an important metric for determining how efficient a model was, so that it can perform efficiently in under-resourced environments. We also chose F1 scores for both the pneumonia and normal class to provide a measurement of both its precision and recall. Across multiple runs on a single model, the only metric that varied was the latency, so an average was reported. Other metrics such as accuracy remained the same across multiple runs on the Raspberry Pi 5.

## Discussion

We observed high accuracies and F1 scores in our results, with some models reaching 0.90+ accuracy while maintaining latencies of less than 1.5 ms, which suggests that our custom-built models maintain high efficiency along with high accuracy. When comparing this to Cococi et al. [8], they achieved a latency of 30 ms for the fastest model on their Raspberry Pi platform, whereas we achieved a latency of 1.46 ms with our fastest model, while maintaining similar accuracies. Moreover, with their 30 ms model, they achieved 0.95 accuracy, whereas our 1.46 ms model achieved 0.91 accuracy. We were also able to build a model with 1.71 ms latency and 0.95 accuracy. This indicates the high performance of our models when only trained on 64 x 64 images, compared to the 224 x 224 images used by Cococi et al. [8].

Furthermore, we are also able to improve the F1 scores compared to Kiche et al. [12]. That study found F1 scores of 0.51 and 0.85 for the normal and pneumonia classes, respectively. In our research, we are able to achieve an F1 score of 0.93 for the normal class and 0.96 for the pneumonia class. By reducing the classes from four to two and developing a custom architecture for the CNN, we are able to improve the F1 scores. 

Additionally, another study [18] also investigated pneumonia classification through machine learning, using a similar amount of images but with three classes (viral pneumonia, bacterial pneumonia, and normal). They used an Xception [19] transfer learning model and achieved a testing accuracy of 0.83. In our work, we used a similar amount of images but only two classes, and were able to achieve a testing accuracy of 0.95 for our Laptop trained model and a 0.91 accuracy for another model. Our results suggest positive performance even when datasets do not have extremely large quantities of images that some hospitals do not have access to.

However, there are limitations to our work. While we simulated under-resourced hospitals having to train the model off-site on a powerful GPU but have to actually diagnose/infer on a less powerful device, it is completely possible that some hospitals and locations will not even have access to a powerful GPU for training. Basically, some hospitals may have to train the entire model and perform inference on-site on a device with very low computing power. Therefore, our research does not account for such cases where hospitals may have to train the model on a lower computing power device. Additionally, while we did see that our latency was much lower compared to previous research by Cococi et al. [8], it is highly likely that a large contribution to the latency drop was the difference in the Raspberry Pi model (Cococi et al. used Raspberry Pi 4 versus 5 in our study). So this latency drop should be interpreted carefully. To investigate these gaps further, future research could include training the models on devices with low computing power rather than just deploying on those devices, and could also compare metrics using the same Raspberry Pi versions or with other ways of keeping the hardware system consistent. Additional research could also be done on classification of more than just two classes, as the decrease in classes may have played a part in the increase of F1 scores compared to earlier research [8].

## Conclusions

Our research suggests that custom-built CNNs are a possibility for efficient classification of pneumonia in resource-limited settings, with high accuracies and low latencies. By bypassing the need for high computing power, high-quality images, and high training image counts, we can provide a way to help hospitals diagnose diseases such as pneumonia more confidently and effectively. While future work could also investigate training with less powerful computers, our work contributes to fixing the problem of inequalities in healthcare, especially with resource gaps across different hospitals and areas.
